# Hierarchical kernel mixture models for the prediction of AIDS disease progression using HIV structural gp120 profiles

**DOI:** 10.1186/1471-2164-11-S4-S22

**Published:** 2010-12-02

**Authors:** Paul D Yoo, Yung Shwen Ho, Jason Ng, Michael Charleston, Nitin K Saksena, Pengyi Yang, Albert Y Zomaya

**Affiliations:** 1 Centre for Distributed and High Performance Computing, University of Sydney, NSW 2006, Australia; 2 Department of Computer Engineering, Khalifa University of Science, Technology and Research (KUSTAR), Abu Dhabi, U.A.E; 3 Retroviral Genetic Laboratory, Centre for Virus Research, Westmead Millennium Institute, Westmead, NSW 2145, Australia; 4 Etisalat British Telecom Innovation Centre (EBTIC), P.O. Box 127788, Abu Dhabi, U.A.E; 5 Centre for Mathematical Biology & Sydney Bioinformatics, University of Sydney, NSW 2006, Australia; 6 National ICT Australia, Australian Technology Park, Eveleigh, NSW 2015, Australia

## Abstract

Changes to the glycosylation profile on HIV gp120 can influence viral pathogenesis and alter AIDS disease progression. The characterization of glycosylation differences at the sequence level is inadequate as the placement of carbohydrates is structurally complex. However, no structural framework is available to date for the study of HIV disease progression. In this study, we propose a novel machine-learning based framework for the prediction of AIDS disease progression in three stages (RP, SP, and LTNP) using the HIV structural gp120 profile. This new intelligent framework proves to be accurate and provides an important benchmark for predicting AIDS disease progression computationally. The model is trained using a novel HIV gp120 glycosylation structural profile to detect possible stages of AIDS disease progression for the target sequences of HIV^+^ individuals. The performance of the proposed model was compared to seven existing different machine-learning models on newly proposed gp120-Benchmark_1 dataset in terms of error-rate (MSE), accuracy (CCI), stability (STD), and complexity (TBM). The novel framework showed better predictive performance with 67.82% CCI, 30.21 MSE, 0.8 STD, and 2.62 TBM on the three stages of AIDS disease progression of 50 HIV+ individuals. This framework is an invaluable bioinformatics tool that will be useful to the clinical assessment of viral pathogenesis.

## Background

The human immunodeficiency virus (HIV) is responsible for the acquired immunodeficiency syndrome (AIDS) disease and 33 million people are infected globally. Infected individuals can live a normal life with drug treatment, but most will eventually progress to AIDS. The duration of disease varies between individuals. Some HIV^+^ patients can progress towards AIDS within two years of primary infection (*rapid progressors* – RP). RP show rapid rise in plasma virus and rapid decline in CD^+^ T cell counts. On the other hand, another group of HIV^+^ patients show steady but gradual increase in viremia and decrease in T cell counts over 10-15 years (*slow progressors* – SP). Only about 1% of HIV^+^ therapy naïve individuals can maintain virus level below detection level, robust T cell counts and experience sustained immune response for more than 20 years (*long term non-progressors* – LTNP). With such a great difference in AIDS disease progression among HIV^+^ patients, much can be learned at the level of differences in viral architecture that exists in HIV variants evolving at different stages of HIV disease and under different immunologic constraints in a given host.

Glycans on the HIV glycoprotein 120 (gp120) surface mask important viral epitopes that host antibodies recognize [1,2], preventing the eradication of the virus. The rapid mutation in gp120 during viral evolution further creates an ever changing landscape of glycosylation patterns of HIV surface glycoprotein gp120 (also known as the “carbohydrate landscape”) that favours host immune evasion. This observation has been termed the glycan shield of HIV [3] and is directly responsible for the persistence of viral infection even after therapy. Thus, any modification to the glycosylation profile of gp120 is likely to affect viral susceptibility to host immune response [4], transmission efficiency [5], infectivity [6] and AIDS disease progression [7]. While the glycosylation of HIV is the main barrier to viral control and eradication, it is possible to harness the protective glycosylation profiles on gp120 against the virus [8] and develop a glycan based approach to vaccine design.

We have previously reported on our findings on glycosylation site interaction within the envelope gp120 [9], which are consistent with the findings by Poon *et al*[10]. The association of multiple glycans within *env* gp120 could be due to the structural placement of the glycosylation sites after protein folding. Glycosylation sites that are far away at the sequence level might actually be close together in three-dimensional (3D) structure of a protein. Thus, the understanding of gp120 glycosylation structural (3D) profile modification can explain the determinants of HIV disease progression. Studies to date have mainly focused on the changes to single glycosylation sites at the sequence level, while the analysis of complete gp120 structural glycan modification is new. This could be due to the lack of an analysis framework for multiple glycan comparison across the entire gp120 sequence.

In this paper, we introduce a novel statistical kernel model, which is designed to learn the complex glycan interactions and predict the differences in AIDS disease progression using the structural 3D glycan profile. It involves the design of semi-parameterized, and support-vector assisted hierarchical mixture model, which is able to effectively capture the information of non-local interactions with strong resistance to vanishing gradient and high-dimensionality problems. The proposed framework successfully classified the changes to glycosylation profiles and segregated HIV disease groups. These results show the utility of new bioinformatics and machine-learning tools in providing useful biological understanding of glycosylation patterns during AIDS disease progression.

## Methods

Our approach to the prediction of AIDS disease progression consists of three consecutive steps: (1) comprehensive HIV dataset construction for the purpose of benchmarking 3D structure-based HIV progress classification methods. (2) novel gp120 structural profile design and (3) the construction of semi-parameterized, and support vector assisted hierarchical mixture model for the exploitation of non-local interaction information from the profile.

### gp120 benchmark dataset

gp120-Benchmark_1 is a newly developed comprehensive dataset for benchmarking 3D structure-based AIDS disease progress classification methods. Based on AIDS progression rates, HIV^+^ individuals have been divided into three categories such as rapid progressors (RP), slow progressors (SP) and long-term non-progressors (LTNP) [11]. RP patients progress towards AIDS within two years of primary infection. RP show rapid rise in plasma virus and rapid decline in CD^+^ T cell counts. SP group of HIV^+^ patients show steady but gradual increase in viremia and decrease in T cell counts over 10-15 years. Only about 1% of HIV^+^ therapy naïve individuals (LTNP) can maintain virus level below detection level, robust T cell counts and experience sustained immune response for more than 20 years [11]. A total of 50 *env* gp120 samples were manually extracted from 50 HIV^+^ individuals, of which 10 samples were LTNPs, 11 were RPs and 29 were SPs. The list of samples with the GenBank accession number, year of sample collection and disease type are shown in Table 1.

**Table 1:** Patient dataset used for structural glycan profiling

Cohort/Country	Sample	GenBank Accession No	Year of sample collection	Disease type
USA	A1	AY835754	1982	RP
USA	A2	AY835765	1984	RP
USA	A3	AY835775	1986	RP
USA	B4	AY835777	1983	RP
USA	B6	AY835778	1986	RP
USA	C7	AY835779	1984	RP
USA	C8	AY835780	1986	RP
USA	D9	AY835781	1983	RP
USA	D10	AY835755	1985	RP
USA	D11	AY835756	1986	RP
USA	E12	AY835757	1986	RP
USA	F1	AY835759	1982	SP
USA	F2	AY835760	1987	SP
USA	F3	AY835761	1991	SP
USA	G4	AY835762	1984	SP
USA	G5	AY835763	1988	SP
USA	G6	AY835764	1992	SP
USA	H7	AY835766	1989	SP
USA	H8	AY835767	1993	SP
USA	J1	AY835769	1985	SP
USA	K4	AY835771	1986	SP
USA	K5	AY835772	1992	SP
USA	K6	AY835773	1994	SP
USA	L7	AY835774	1986	SP
USA	M1	AY835748	1983	LTNP
USA	M2	AY835749	1986	LTNP
USA	M3	AY835750	1989	LTNP
USA	M4	AY835751	1990	LTNP
USA	M5	AY835752	1992	LTNP
USA	N8	AY835753	1996	LTNP
Canada	CAN_A_1	AY779564	1996	LTNP
Canada	CAN_A_3	AY779550	1998	LTNP
Canada	CAN_A_5	AY779551	1999	LTNP
Canada	CAN_A_6	AY779552	2000	LTNP
Canada	CAN_B_3	AY779553	1994	SP
Canada	CAN_B_4	AY779554	1997	SP
Canada	CAN_B_5	AY779555	1998	SP
Canada	CAN_B_6	AY779556	1999	SP
Canada	CAN_C_2	AY779557	1992	SP
Canada	CAN_C_3	AY779558	1993	SP
Canada	CAN_C_5	AY779559	1994	SP
Canada	CAN_C_6	AY779560	1994	SP
Canada	CAN_C_8	AY779561	1996	SP
Canada	CAN_C_10	AY779562	1998	SP
Australia	1181	GQ995529	1995	SP
Australia	1182	GQ995528	1995	SP
Australia	BB_76	GQ995532	1982	SP
Australia	BB_24	GQ995530	1984	SP
Australia	BB_42	GQ995531	1984	SP
Australia	BB_92	GQ995533	1983	SP

gp120-Benchmark_1 is available at http://www.cs.usyd.edu.au/~yangpy/software/AIDS-progression.html

### Structural gp120 profiling

**Figure 1:**
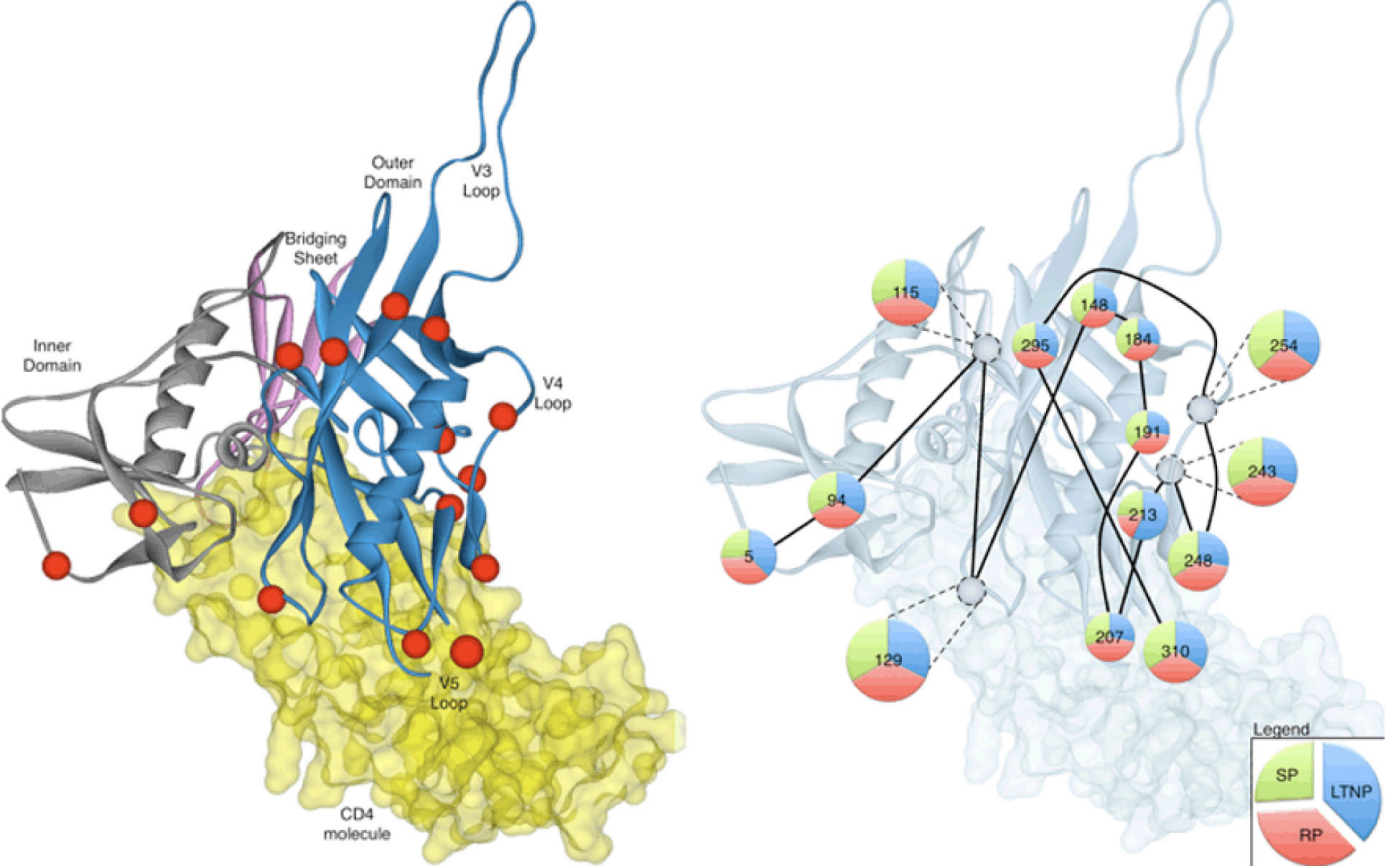
Annotation of the glycans on the template *env* gp120 crystal structure PB4C (left-hand), and variations in structural locations of glycans (right-hand).

The glycosylation of HIV occurs on the asparagine residue of a NX[ST] motif where X can be any amino acid residue except proline [35,36]. To create a whole envelope glycan profile, the 3D coordinates of the asparagine residue from every glycosylation site were extracted from the gp120 structure models. The extracted glycans from the query model were matched to an appropriate glycosylation site from the template model (Figure 1, left-hand). Mutations, insertions and deletions (indels) are common within the gp120 gene and can thus complicate the matching of target and template glycosylation sites. Further, it is also difficult to differentiate whether a glycosylation site has moved if the neighbouring glycans are clustered close together. To overcome these problems, we developed a protocol to loosely encapsulate the movement, insertion and deletion of glycosylation sites in the HIV envelope gp120 models, to facilitate the glycan matching process. First, the glycosylation sites were separated into their respective regions (V1, V2, C2, V3, C3, V4, C4, V5, C5). The glycan from the query model can only be matched with the template glycan of the same region. Second, within each region, a distance matrix was created by calculating the 3D distance between every glycan of the query and template models. Finally, every glycan in the query model was matched to the closest glycan from the target model and their distance noted.

The addition of glycosylation sites as compared to the template model is expressed as an additional distance value in the glycosylation profile. The movement of glycosylation sites is reflected as a significant change in distance value in the profile, while retaining its association with the same template glycan. Finally, the deletion of glycans was coded as an absent distance value in the profile as compared to other models. The glycan profile reflects the variation in the global glycosylation pattern in relation to the given template model. The glycan profiles for all the sequences in our dataset are compiled in an N ? M matrix, where N is the maximum number of glycans in our dataset and M is the number of sequences in the dataset. Sequences with fewer glycosylation sites were padded with a distance value of zero on either side of the matrix.

### Nonlocal interactions in sequence and vanishing gradient problem

A machine learning framework with kernel method [12] was used to learn and characterize the changes to glycosylation profiles for the prediction of HIV disease progression. Existing large machine-learning algorithms like neural networks have performed well in the analysis of sequence-based problems, where the information is processed in the order that they are given. However, these were not designed to analyse the long-range dependencies between glycosylation sites at the sequence level. This is due to the lack of an efficient algorithm for numerical optimization, commonly known as the vanishing-gradient problem [13]. For example, when the glycosylation sites are analysed in the order they appear in the sequence, the dependency between a glycan X and a previously processed glycan Y might not be learnt. If glycan X and glycan Y are processed immediately after one another (short range), their dependency can be learnt. However, if there are multiple glycans between X and Y (long range), the neural network can only learn the dependencies between glycan X and the sum of all glycan information before X (including glycan Y). While the dependencies between X and Y are somewhat established because of the partial inclusion of glycan Y in the combined information, the long range dependency learned between glycans X and Y are not sufficient to classify HIV disease progression. Furthermore, error minimization is known to fail in the presence of dependences that are far apart in the sequence space [14,15].

### Support vector assisted hierarchical mixture model (SV-HMM)

One possible solution to this problem is to ameliorate the vanishing gradient problem by providing the kernel function with information of distantly located glycan. This gives the machine-learning framework a fair chance of learning any long-range interaction between glycosylation sites. We developed a hierarchical kernel mixture model that combines a modular approach with local linear support vector classifiers, which can effectively analyse distantly related information.

**Figure 2:**
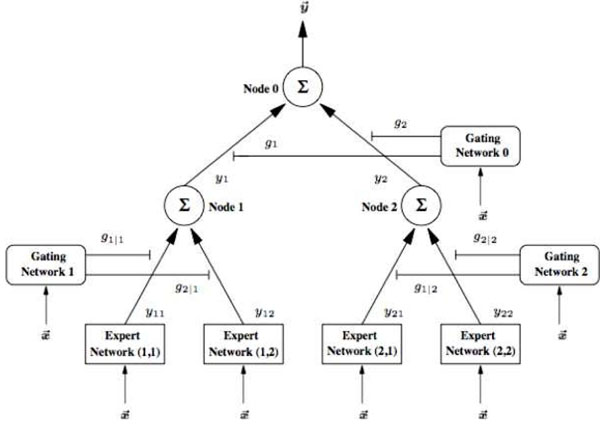
**Modular hierarchical kernel experts as a global model.** HME has a tree-like structure that uses the divide and conquer principle to learn the interactions between HIV glycosylation sites. At the bottom of the tree (leaf nodes) are multiple locality effective support vector, that will analyse the similarities between the given glycosylation sites.

At the top level of our framework, we have adopted a well-known Hierarchical Mixture of Experts (HME) model for regression and classification [16]. The HME model is a tree-like structure (Figure 2) that uses the divide and conquer principle to learn the interactions between HIV glycosylation sites. At the bottom of the tree (leaf nodes) are multiple locality effective support vector, that will analyse the similarities between the given glycosylation sites.

HME describes a conditional probability distribution over a vector *t* of target variables, conditioned on a vector *x* of inputs. Consider the case of functional mapping learning of the type  based on training data set *T =* (*x*^(^*^t^*^)^*, y*^(^*^t^*^)^)*, t = 0, …, n* with  and a corresponding desired response . All of the networks, both experts and gating, receive the same input vector at the *t^th^* time instant, *x*^(^*^t^*^)^. However, while the gating networks use this input to compute confidence level values for the outputs of the connected expert networks, the expert networks themselves use the input to generate an estimate of the desired output value. The outputs of the gating networks are scalar values and are a partition of unity at each point in the input space, i.e. a probability set. Thus, consider a two-layered binary branching HME: Each of the expert local models (*i, j*) produces outputs *y_ij_* from the input vector *x*^(^*^t^*^)^ according to the relationship: , where *f* is a neural-network mapping using input *x*^(^*^t^*^)^ and its corresponding weight matrix . The outputs of the gating network *g_i_* at the top level are computed according to:

where *V_i_* is the weight vector associated with gating network *g_i_*. Due to the special form of the softmax being non-linear, the *g_i_*’s are positive and sum up to one for each input vector *x*^(^*^t^*^)^*.* The lower level gating networks compute their output activations similar to the top level gating network according to the following expression:

The output activations of the expert networks are weighted by the gating networks’ output activations as they proceed up the tree to form the overall output vector. Specifically, the output of the *i^th^* internal node in the second layer of the tree is:

while the output at the top level node is:

Since both the *g*’s and the *y*’s depend on the input *x*^(^*^t^*^)^, the overall output of the architecture is a non-linear function of the input.

### Local kernel machine as a collaborating filter to SV-HMM

As a key local collaborator, we use a support-vector classifier [17] to assist the global HME model. SVMs are known as maximum margin classifiers since they classify their objects by minimizing the empirical generalization error and maximizing the geometric margin simultaneously. Where the two classes are not separable, they map the input space into a high-dimensional feature space (where the classes are linearly separable) by using a non-linear kernel function. The kernel function calculates the scalar product of the images of two examples in the feature space. Given a *n*-dimensional input vector, *x_i_=*(*x_1_,x_2_,…,x_n_*) with class labels, *y_i_* ∈ {+1,–1}, (*i*=1,2,...,*N*)*,* the hyperplane decision function of binary SVM with kernel method is written as:

and the following quadratic program:

maximize 

subject to *a_i_* ≥ 0,  and 

where  is the number of training patters; *a_i_* are the parameters of the SVM;  is a suitable kernel function, and *b* is the bias term.

### Semi-parametric modelling to the local kernels

The use of *x_i_* examples, especially in high-dimensional space causes several key problems. First, the good data fitting capacity of the flexible “model-free” approach often tends to fit the training data very well and thus, have a low bias. However, the potential risk is overfitting that causes high variance in generalisation. In general, the variance is shown to be a more important factor than the learning bias in poor prediction performance [18]. Second, with the high-dimensional data such as proteins, as the number of hidden nodes of the network is severely increased, it eventually leads to an exponential rise in computational complexity. A high complexity model generally shows a low bias but a high variance [19]. On the other hand, a model with low complexity shows a high bias but a low variance. Hence, a good model should balance well between model bias and model variance. This problem is generally regarded as the term *bias-variance tradeoff*.

One of the solutions to the above problem is the so-called semi-parametric modeling. Semi-parametric models take assumptions that are stronger than those of non-parametric models, but are less restrictive than those of parametric model. In particular, they avoid many serious practical disadvantages of non-parametric methods at the price of an increased risk of specification errors.

**Figure 3:**
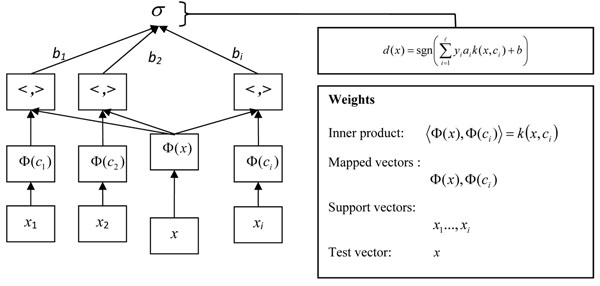
**The basic architecture of semi-parameterized SV-based local models.** The centroid vectors from voronoi region for each training sample *x_i_* used in the SVM decision function. SVM is considered as a purely non-parametric model, whereas SV-HMM is considered as semi-parametric model as it adopts the method of grouping the associated input vectors in each class *i*. RBF kernel has been used for the SV-based local models.

To semi-parameterize SV-HMM, we substitute the centroid vectors from *voronoi* region [20] for each training sample *x_i_* used in the SVM decision function (Figure 3). Consider a training sequence consisting of *M* source vectors, *T=*{*x_1_, x_2_, …,x_m_*}. *M* is assumed to be sufficiently large and so that all the statistical properties of the source are captured by the training sequence. We assume that the source vectors are *k*-dimensional, *X_m_=*(*x_m,1_, x_m,2_, …, x_m,k_*)*, m=1,2,…,M*. These vectors are compressed by choosing the nearest matching vectors and form a codebook consisting the set of all the code-vectors. *N* is the number of code-vectors, *C=*{*c_1_,c_2_,…,c_n_*} and each code-vector is *k*-dimensional, *c_n_=*(*c_n,1_,c_n,2_,…,c_n,k_*)*,n=1,2,…,N*. The representative codevector is determined to be the closest in Euclidean distance from the source vector. The Euclidean distance is defined by:

where *x_j_* is the *j^th^* component of the source vector, and *c_ij_* is the *j^th^* is components of the code-vector *c_i_*. *S_n_* is the nearest-neighbor region associated with code-vector *c_n_*, and the partitions of the whole region are denoted by *P=*{*S_1_,S_2_,…,S_N_*}. If the source vector *X_m_* is in the region *S_n_*, its approximation can be denoted by *Q*(*X_m_*)*=c_n_,* if *X_m_* ∈ *S_n_*. The *Voronoi* region is defined by:

To find the most optimal *C* and *P*, vector quantization uses a square-error distortion measure specifying exactly how close the approximation is. The distortion measure can be given as:

If *C* and *P* are solution to the above minimization problem, then it must satisfy two conditions namely nearest neighbor and centroid conditions. The nearest neighbor condition indicates the sub-region *S_n_* should consist of all vectors that are closer to *c_n_* than any of the other code-vectors. It is written as:

The centroid condition requires the code-vector *c_n_* should be average of all those training vectors that are in its *Voronoi Region S_n_.*

The centroid vector of each *voronoi* region which can be expressed as:

The new local SVM’s approximation can be written as:

and the following quadratic program:

maximize 

subject to *a_i_* ≥ 0,  and 

SVM is considered as a purely non-parametric model, whereas SV-HMM is considered as semi-parametric model as it adopts the method of grouping the associated input vectors in each class *i*. Hence, the performance of the proposed model has some advantages in comparison to both pure parametric models and pure non-parametric models in terms of learning bias and generalization variance especially on high-dimensional protein datasets.

### Prediction of AIDS disease progression

Our hierarchical mixture modeling contains a number of consecutive steps. First, structural glycosylation profiles from our HIV^+^ RP, SP and LTNP sequences were used as the input data in our computational kernel analysis. By learning the differences in glycosylation changes between patient samples, the SV-HMM was used to classify a previously unseen glycosylation profile into RPs, SPs or LTNPs. Second, the predicted classifications were compared with the true disease group for the glycosylation profile. These experiments were performed in a series of steps. The SV-HMM and seven other well-regarded machine learning models, transductive support vector machine (SVM_LIB_) [21], SVM based decorate model (Decorate _SVM_) [22], multi-layered perceptron (MLP) [23], radial basis function network (RBFN) [24], logistics [25], and decision trees (J48) [26] – were used to analyse the glycosylation profiles.

**Figure 4:**
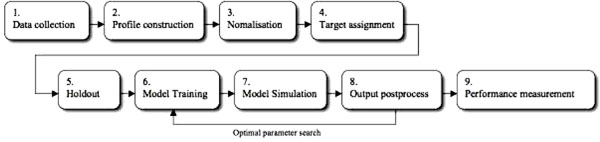
**The flowchart of SV-HMM showing the stepwise procedure.** The above figure shows the stepwise procedure we have performed. (1) data collection, building gp120 benchmark dataset and pre-processing datasets; (2) structural gp120 profile construction including matching up and calculating the 3D distance between every glycan of the query and template models. (3) the information obtained in (2) and (3) were combined and normalised to fall in the interval [--1, 1] to be fed into networks; (4) target levels were assigned to each profile (positive, +1, for RPs, 0 for SP. –1 for LTNP); (5) a hold-out method, to divide the combined dataset into ten subsets (training and testing sets); (6) model training on each set, to create a model; (7) simulation of each model on the test set, to obtain predicted outputs; and (8) post-processing to find predicted HIV progressor groups. The procedure from (6) to (8) was performed iteratively until we obtained the most suitable kernel and the optimal hyperparameters for SV-HMM for gp120 benchmark dataset.

For the analysis of each kernel model, we performed a tenfold cross-validation evaluation on the dataset – most widely adopted method for fair model evaluation of general computational sequence-based classification [37-42]. During the cross-validation phase, one fold data were randomly chosen and excluded from the training set before model learning began, and were later used to test the performance of the learnt model. This process was repeated ten times. The cross validation protocol was chosen to solve the potential problems caused by residual evaluations. This is because, if the entire dataset were used for training, the model could not provide adequate indication of its effectiveness in predicting any unseen data. Thus, it does not matter how the data is divided as every data point gets to be in the test set exactly once and, in the training sets, nine out of ten times. Since every data point is tested exactly once, it has been a widely used evaluation method for real experimental sequence data, (just as the case in gp120 benchmark experiment) [37-42]. Finally, each model has been measured by the predictive accuracy (CCI: correctly classified instances) of the kernel model. The final CCI value was calculated based on the average of all the prediction accuracies observed during the tenfold validation process. The mean squared error (MSE: a quantity used to measure how close forecasts or predictions are to the eventual outcomes), time (TBM: time to build model), and model stability (STD: ST Deviation obtained from ten sub-samples) were also measured in the experiments. The stepwise procedure has been provided in Figure 4.

## Results

In our comparison between the eight machine-learning models, SVM-assisted hierarchical mixture models (SV-HMM: semi-parametric, HME_SVM_: non-parametric) were shown to be most suitable (CCI: 67.82%, 69.38%) for the classification of HIV disease types using the glycosylation profiles (Table 2). In general, SVM-based models such as SV-HMM, HME_SVM_, SVM_LIB_ and Decorate_SVM_ performed better than the other models in terms of CCI. This suggests that the linear support vector approaches with RBF kernel used in the standard SVMmodels accurately learns the characteristics from structural gp120 profiles for the prediction of AIDS disease progression. Mean testing data ±STD obtained by ANOVA test using optimal settings for each model. The ANOVA CCI and STD of each model are insignificant, which suggest that all the models are performing consistently in the experiment. The performance of our semi-parameterized SV-MHH model was the most stable (STD: 0.8) in the classification of HIV disease stages than the other machine-learning models, with the exception of the Logistic model, which also gave good prediction stability due to its statistical nature.

**Table 2:** Model comparison on gp120_Benchmark_1 dataset

Models	MSE_RP_	MSE_SP_	MSE_LTNP_	MSE_Overall_	CCI	STD	TBM
SV-HMM	38.02	30.46	45.57	30.21	67.82	0.8	2.62
HME_SVM_	36.35	24.13	44.43	29.76	69.38	1.9	1.35
SVM_LIB_	54.53	20.68	44.43	32.65	67.39	1.1	0.17
Decorate_SVM_	81.82	10.34	55.55	32.88	65.31	2.7	3.02
MLP	72.43	27.59	55.57	27.27	57.14	4.2	4.14
RBFN	63.63	27.58	77.78	29.32	55.10	3.8	0.06
Logistic	63.63	34.48	66.66	31.35	53.06	1.0	0.11
J48	90.91	37.93	66.67	37.77	44.90	3.0	0.01

The final CCI value was calculated based on the average of all the prediction accuracies ob-served during the tenfold validation process. MSE, TBM and STD (ST Deviation obtained from ten sub-samples) indicate the accuracy, complexity and stability of the model respectively.

These results showed that the hierarchical mixture model approach used in the proposed SV-HMM model can consistently capture critical changes to HIV glycosylation profiles and can be used to predict AIDS disease progression. Associative differences in the glycosylation profile that determine disease progression might be located far apart in the sequence, but close together in the tertiary structure. The results also suggest that the modular approaches used in SV-HMM and HME_SVM_ is able to capture these non-local interactions. Thus, our proposed models are resistant to model over-fitting and weak signal to noise ratio during the learning phase and can avoid the vanishing gradient problem in this scenario.

Our structural glycosylation profiles of our gp120 models were created using the template crystal structure. The distance between the matching glycosylation sites from our model to the template structure (Figure 1, left-hand) was calculated to create the glycan profile. Most glycosylation sites had an equal contribution of structural variations by the three disease groups, with the exception of glycan N213 in the C3 region, where there were more structural glycan differences in the LTNP models (Figure 1, right-hand). When we compared the average structural variation observed, glycan sites 115, 129, 243, 254 were more structurally variable (>50%).

The gp120 protein exists as a trimer and the inner domain of gp120 (coloured grey) faces the trimer axis (left-hand). The outer domain (coloured blue) faces away from the trimer axis and is exposed on the envelope surface. Coloured red spheres represent the glycosylation sites on our template structure. The V3, V4 and V5 loop regions are annotated. The CD4 molecule (yellow) that binds to gp120 is included to give a better perspective of the protein. Each pie chart (right-hand) represents the percentage of glycan structural variations by the LTNP, RP or SP samples at each glycosylation site. The area of the pie charts is proportional to average structural variation observed. Connecting lines indicate the primary (sequence) ordering of the glycosylation sites.

## Discussion

A prediction accuracy of 67 to 69% was achieved in the classification of HIV envelope sequences from RP, SP and LTNP patients. The structural glycan profiling technique was designed to encapsulate the frequent and varied structural changes to glycosylation sites observed in HIV envelope gp120. Structural glycan modifications from the entire envelope gp120 were characterized at the same time to provide a macroscopic view of the carbohydrate landscape. Interactions between glycosylation sites were analysed by the division of the glycan input space into smaller and more manageable problems, using the modular kernel design adopted in hierarchical kernel mixture architecture. These partial solutions were then integrated to yield an overall solution to the whole problem. The modular approach enforces constant error flow through the internal states of neural-network units. By doing so, we were able to study interactions between glycosylation sites that are located far apart in the sequence, which were not possible using traditional machine-learning methods. While the framework was successful in identifying critical changes to glycan profiles for different disease progression rates, we were limited to the number of available patient samples that were well characterized clinically. Having more SP sequences in our dataset also meant that we had to address the potential bias in our learning model.

Another reason for the reduction of predictive power for HIV disease progression could be due to the absence of structural information from the V1-V2 loops of gp120. During the crystallization process, the V1-V2 loops were normally deleted in order to promote better crystallization. Thus, no V1-V2 structural information is available to date. Unfortunately, the V1 and V2 glycans are important to disease progression. Structurally, the V1 and V2 glycans protect the bridging sheet between the inner and outer domain of HIV envelope gp120 [27], and influence AIDS progression through host-receptor modulation during an infection. Any changes to the V2 glycan can potentially restrict the capacity of HIV-1 to replicate [28]. Without the correct glycosylation profile from the V1 V2 loops, we can only, at best, develop a partial mapping of the envelope carbohydrate landscape to determine AIDS progression.

During the glycan profiling, the V3 region of our sequence was not included in the analyses, as the template crystal structure (PDB: 2B4C) was not glycosylated in the V3 region. This might also have partially disadvantaged the model’s prediction power, as the V3 region is known to indirectly influence disease progression rates. When HIV infects a host cell, the gp120 protein first attaches to a CD4 molecule, followed by the binding to either the CCR5 or CXCR4 co receptor [29,30]. Viral strains that preferentially bind to CCR5 are less pathogenic, while viruses that prefer CXCR4 are more virulent and are associated with faster disease progression [31]. The ability to use the CXCR4 co-receptor is influenced by the absence of the glycan in the V3 region only [32], whereas the use of CCR5 co-receptor is collectively influenced by the amino acid makeup and glycan profiles in the V1, V2 and V3 regions all together [32,33]. Thus, the occlusion of V3 glycans from our study might have partially weakened our prediction results. On the contrary, the stable prediction rate without the influence of V1, V2 and V3 glycans showed that the other glycans outside these regions are important to disease progression. This observation is consistent with several previously reported findings. For example, glycan modification within the C3 region can affect viral fusogenicity and entry kinetics [6], while both V4 [34] and C4 [7] glycans can affect the CD4 binding.

## Conclusion

This paper addressed two important issues in the prediction of AIDS disease progression. First, it provides a formal framework for researchers to understand the effect of whole envelope structural glycan modification against phenotypic changes to viral pathogenesis, like receptor binding ability, replication efficiency and infectivity predictions. It is the first study in the literature that uses the structural glycan information in the analysis of HIV which was made possible by the availability of the HIV envelope crystal structures and sophisticated homology modelling protocols. Second, our novel framework uses semi-parameterized, support-vector assisted hierarchical mixture model, which is able to effectively exploit the information of non-local interactions with strong resistance to vanishing-gradient and high-dimensionality problems. This also provided a way of fine-tuning the model by the adjustment of hyper-parameters as well as providing efficient semi-parametric approximation. With the newly built gp120-Benchmark_1 dataset, our novel framework which uses SV-HMM and HIV structural gp120 profile has set an important benchmark for the computational prediction of AIDS disease progression.

## List of abbreviation used

AIDS: Acquired Immune Deficiency Syndrome; ANOVA: The Analysis Of Variance; CCI: Correctly Classified Instances); HIV: Human Immunodeficiency Virus; HKMM: Hierarchical Kernel Mixture Model; HME: Hierarchical Mixture of Experts; J48: Decision Trees; MLP: Multi-Layered Perceptron; PDB: Protein Data Bank; RBFN: Radial Basis Function Network; RP: Rapid Progressors; SP: Slow Progressors; STD: ST Deviation; STNP: Long Term Non-Progressors; SVM: Support Vector Machine; TBM: Time to Build Model.

## Competing interests

The authors declare that there is no competing interests.

## Authors’ contributions

PDY developed and implemented the new kernel model (HKMM). YSH and PDY developed glycol-profile and gp120 dataset, and drafted the manuscript. YSH, JN, PY interpreted the results with PDY. NKS, MC and AYZ edited the manuscript and offered much advices and insight during the project.

## References

[B1] Wyatt R Kwong PD Desjardins E Sweet RW Robinson J Hendrickson WA Sodroski JG The antigenic structure of the HIV gp120 envelope glycoprotein Nature 1998 393 6686 705 11 10.1038/31514 9641684

[B2] Kwong PD Wyatt R Sattentau QJ Sodroski J Hendrickson WA Oligomeric modeling and electrostatic analysis of the gp120 envelope glycoprotein of human immunodeficiency virus J Virol 2000 74 4 1961 72 10.1128/JVI.74.4.1961-1972.2000 10644369 PMC111674

[B3] Wei X Decker JM Wang S Hui H Kappes JC Wu X Salazar-Gonzalez JF Salazar MG Kilby JM Saag MS Komarova NL Nowak MA Hahn BH Kwong PD Shaw GM Antibody neutralization and escape by HIV-1 Nature 2003 422 6929 307 12 10.1038/nature01470 12646921

[B4] Sagar M Wu X Lee S Overbaugh J Human immunodeficiency virus type 1 V1-V2 envelope loop sequences expand and add glycosylation sites over the course of infection and these modifications affect antibody neutralization sensitivity J Virol 2006 80 19 9586 98 10.1128/JVI.00141-06 16973562 PMC1617272

[B5] Chohan B Lang D Sagar M Korber B Lavreys L Richardson B Overbaugh J Selection for human immunodeficiency virus type 1 envelope glycosylation variants with shorter V1-V2 loop sequences occurs during transmission of certain genetic subtypes and may impact viral RNA levels J Virol 2005 79 10 6528 31 10.1128/JVI.79.10.6528-6531.2005 15858037 PMC1091724

[B6] Sterjovski J Churchill MJ Ellett A Gray LR Roche MJ Dunfee RL Purcell DF Saksena NK Wang B Sonza S Wesselingh SL Karlsson I Fenyo EM Gabuzda D Cunningham AL Gorry PR Asn 362 in gp120 contributes to enhanced fusogenicity by CCR5-restricted HIV-1 envelope glycoprotein variants from patients with AIDS Retrovirology 2007 4 89 10.1186/1742-4690-4-89 18076768 PMC2225424

[B7] Li H Xu CF Blais S Wan Q Zhang HT Landry SJ Hioe CE Proximal glycans outside of the epitopes regulate the presentation of HIV-1 envelope gp120 helper epitopes J Immunol 2009 182 10 6369 78 10.4049/jimmunol.0804287 19414790 PMC2808118

[B8] Scanlan CN Offer J Zitzmann N Dwek RA Exploiting the defensive sugars of HIV-1 for drug and vaccine design Nature 2007 446 7139 1038 45 10.1038/nature05818 17460665

[B9] Ho YS Abecasis AB Theys K Deforche K Dwyer DE Charleston M Vandamme AM Saksena NK HIV-1 gp120 N-linked glycosylation differs between plasma and leukocyte compartments Virol J 2008 5 14 10.1186/1743-422X-5-14 18215327 PMC2265691

[B10] Poon AF Lewis FI Pond SL Frost SD Evolutionary interactions between N-linked glycosylation sites in the HIV-1 envelope PLoS Comput Bio 2007 13 1 e11 10.1371/journal.pcbi.0030011 PMC1779302 17238283

[B11] Saksena NK Rodes B Wang B Soriano V Elite HIV controllers: myth or reality? AIDS Rev 2007 9 4 195 207 18219363

[B12] Ben-Hur A Ong CS Sonnenburg S Scholkopf B Ratsch G Support vector machines and kernels for computational biology PLoS Comput Biol 2008 4 10 e1000173 10.1371/journal.pcbi.1000173 18974822 PMC2547983

[B13] Hochreiter S The vanishing gradient problem during learning recurrent neural nets and problem solutions International Journal of Uncertainty Fuzziness and Knowledge-Based Systems 1998 6 2 107 116 10.1142/S0218488598000094

[B14] Hochreiter S Informatik F Bengio Y Frasconi P Schmidhuber J Kolen J Kremer S Gradient Flow in Recurrent Nets: the Difficulty of Learning Long-Term Dependencies In Field Guide to Dynamical Recurrent Networks IEEE 2000 in press

[B15] Bengio Y Simard P Frasconi P Learning long-term dependencies with gradient descent is difficult IEEE Trans Neural Netw 1994 5 2 157 66 10.1109/72.279181 18267787

[B16] Jordan MI Jacobs RA Hierarchical mixtures of experts and the EM algorithm Neural Comp 1994 6 181 214 10.1162/neco.1994.6.2.181

[B17] Vapnik V The Nature of Statistical Learning Theory 1995 Springer-Verlag8555380

[B18] Dietterich TG Bakiri G Machine Learning bias statistical bias and statistical variance of decision tree algorithms Dept 1995

[B19] Larose DT Discovering Knowledge in Data Wiley 2005

[B20] Okabe A Boots B Sugihara K Chiu SN Spatial Tessellations - Concepts and Applications of Voronoi Diagrams John Wiley 2000 2 671

[B21] Joachims T Transductive Inference for Text Classification using Support Vector Machines In International Conference on Machine Learning (ICML), Bled, Slovenia 1999

[B22] Melville P Mooney RJ In Constructing Diverse Classifier Ensembles using Artificial Training Examples IJACI, Acapulco, Mexico 2003 505 510

[B23] Haykin S Neural Networks: A Comprehensive Foundation 1998 2 Prentice Hall9697135

[B24] Yee P Haykin S Regularized Radial Basis Function Networks: Theory and Applications 2001 John Wiley & Sons: Canada

[B25] Bishop CM Pattern Recognition and Machine Learning (Information Science and Statistics) 2007 Springer: Cambrridge UK

[B26] Quinlan JR C4.5: Programs for Machine Learning Morgan Kaufmann 1992

[B27] Kwong PD Wyatt R Robinson J Sweet RW Sodroski J Hendrickson WA Structure of an HIV gp120 envelope glycoprotein in complex with the CD4 receptor and a neutralizing human antibody Nature 1998 393 6686 648 59 10.1038/31405 9641677 PMC5629912

[B28] Shioda T Oka S Xin X Liu H Harukuni R Kurotani A Fukushima M Hasan MK Shiino T Takebe Y Iwamoto A Nagai Y In vivo sequence variability of human immunodeficiency virus type 1 envelope gp120: association of V2 extension with slow disease progression J Virol 1997 71 7 4871 81 9188549 10.1128/jvi.71.7.4871-4881.1997 PMC191717

[B29] Berger EA HIV entry and tropism: the chemokine receptor connection AIDS 1997 11 SupplA S3 16 9451961

[B30] Zhang YJ Moore JP Will multiple coreceptors need to be targeted by inhibitors of human immunodeficiency virus type 1 entry? J Virol 1999 73 4 3443 8 10074200 10.1128/jvi.73.4.3443-3448.1999 PMC104110

[B31] Masciotra S Owen SM Rudolph D Yang C Wang B Saksena N Spira T Dhawan S Lal RB Temporal relationship between V1V2 variation macrophage replication and coreceptor adaptation during HIV-1 disease progression AIDS 2002 16 14 1887 98 10.1097/00002030-200209270-00005 12351948

[B32] Nabatov AA Pollakis G Linnemann T Kliphius A Chalaby MI Paxton WA Intrapatient alterations in the human immunodeficiency virus type 1 gp120 V1V2 and V3 regions differentially modulate coreceptor usage virus inhibition by CC/CXC chemokines soluble CD4 and the b12 and 2G12 monoclonal antibodies J Virol 2004 78 1 524 30 10.1128/JVI.78.1.524-530.2004 14671134 PMC303404

[B33] Ly A Stamatatos L V2 loop glycosylation of the human immunodeficiency virus type 1 SF162 envelope facilitates interaction of this protein with CD4 and CCR5 receptors and protects the virus from neutralization by anti-V3 loop and anti-CD4 binding site antibodies J Virol 2000 74 15 6769 76 10.1128/JVI.74.15.6769-6776.2000 10888615 PMC112193

[B34] Pantophlet R Ollmann E Saphire Poignard P Parren PW Wilson IA Burton DR Fine mapping of the interaction of neutralizing and nonneutralizing monoclonal antibodies with the CD4 binding site of human immunodeficiency virus type 1 gp120 J Virol 2003 77 1 642 58 10.1128/JVI.77.1.642-658.2003 12477867 PMC140633

[B35] Ben-dor S Esterman N Biases and complex patterns in the residues flanking protein N-glycosylation sites Glycobiology 2004 14 95 101 10.1093/glycob/cwh004 14514714

[B36] Shakin-Eshleman SH Spitalnik SL: et al: The amino acid at the X position of an Asn-X-Ser sequon is an important determinant of N-linked core-glycosylation efficiency J Biol Chem 1996 271 6363 6 10.1074/jbc.271.11.6363 8626433

[B37] Yoo PD Ho S Zhou BB Zomaya AY SiteSeek: Protein Post-Translational Modification Analysis Using Adaptive Locality-Effective Kernel Methods and New Profiles BMC Bioinformatics 2008 9 272 10.1186/1471-2105-9-272 18541042 PMC2442102

[B38] Baldi P Brunak S Bioinformatics: The machine learning approach 2nd ed MIT Press 2001 Cambridge Mass 2

[B39] Jones DT Protein secondary structure prediction based on position-specific scoring matrices J Molecular Biology 1999 292 2 195 202 10.1006/jmbi.1999.3091 10493868

[B40] Ginalski K Elofsson A Fischer D Rychlewski L 3D-Jury: a simple approach to improve protein structure predictions Bioinformatics 2003 19 8 1015 1018 10.1093/bioinformatics/btg124 12761065

[B41] Sim J Kim SY Lee J PRODO: Prediction of Protein Domain Boundaries using Neural Networks Proteins 2005 59 627 632 10.1002/prot.20442 15789433

[B42] Sikder AR Zomaya AY Improving the performance of DomainDiscovery of protein domain boundary assignment using inter-domain linker index BMC Bioinformatics 2006 7 5 S6 10.1186/1471-2105-7-S5-S6 17254311 PMC1764483

